# Stem Cell Therapy in Patients with Chronic Nonischemic Heart Failure

**DOI:** 10.1155/2018/6487812

**Published:** 2018-01-18

**Authors:** Gregor Poglajen, Gregor Zemljič, Sabina Frljak, Andraž Cerar, Vesna Andročec, Matjaž Sever, Peter Černelč

**Affiliations:** ^1^Advanced Heart Failure and Transplantation Center, University Medical Center Ljubljana, Ljubljana, Slovenia; ^2^Department of Hematology, University Medical Center Ljubljana, Ljubljana, Slovenia

## Abstract

**Aim of the Review:**

The aim of this review is to discuss recent advances in clinical aspects of stem cell therapy in chronic nonischemic heart failure (DCMP) with emphasis on patient selection, stem cell types, and delivery methods.

**Recent Findings:**

Several stem cell types have been considered for the treatment of DCMP patients. Bone marrow-derived cells and CD34^+^ cells have been demonstrated to improve myocardial performance, functional capacity, and neurohumoral activation. Furthermore, allogeneic mesenchymal stem cells were also shown to be effective in improving heart function in this patient population; this may represent an important step towards the development of a standardized stem cell product for widespread clinical use in patients with DCMP.

**Summary:**

The trials of stem cell therapy in DCMP patients have shown some promising results, thus making DCMP apparently more inviting target for stem cell therapy than chronic ischemic heart failure, where studies to date failed to demonstrate a consistent effect of stem cells on myocardial performance. Future stem cell strategies should aim for more personalized therapeutic approach by establishing the optimal stem cell type or their combination, dose, and delivery method for an individual patient adjusted for patient's age and stage of the disease.

## 1. Introduction

Despite significant advances in medical and device therapy over the past decades, chronic heart failure (HF) represents an increasingly common and debilitating disorder worldwide. Even in the 21st century, HF carries a dismal prognosis and is related to unacceptably high mortality rates [1]. Although current HF treatment options have been shown to improve HF symptoms and signs, to reduce heart failure-related hospital admissions, and above all to improve patients' survival [2], no therapeutic modality to date has addressed the repair of damaged/lost myocardial tissue. The latter represents the central common underlying pathophysiologic mechanisms of heart failure development and progression regardless of the initial trigger of myocardial damage. Ever since the first successful use of bone marrow stem cells in an experimental model of acute myocardial infarction was published [3], there has been a growing interest in the HF community to apply stem cell therapy for the treatment of chronic ischemic HF. Although, especially in the younger (<70 years) population of heart failure patients, chronic nonischemic heart failure (DCMP) has become the most prevalent form of the disease leading to heart transplantation [4], it has gained surprisingly little attention in the field of stem cell therapy with only a handful of studies addressing this steadily increasing pool of patients (Table 1).

Thus, the aim of this review is to discuss current status and recent advances of stem cell therapy in the treatment of DCMP, with emphasis on patient selection, stem cell types, and clinical efficiency of this treatment modality.

## 2. Pathophysiologic Basis of Nonischemic Heart Failure and the Significance of Microvascular Ischemia

DCMP is characterized by left or biventricular dilation with concomitant systolic dysfunction in the absence of extrinsic factors that may cause a similar phenotype such as coronary artery disease, arterial hypertension, valvular heart disease, or congenital heart disease. This disorder can develop at any age and regardless of gender or race [5]. The etiology of DCMP is multifactorial, and currently, it is believed that it occurs due to the interplay between genetic factors, infectious (mainly viral) causes, mechanical stress, and toxicity-related causes [5]. Macroscopic enlargement of all four chambers with relatively more pronounced dilation of the ventricles than the atria is seen. Histological examination of the ventricular myocardium occasionally shows areas of cardiomyocyte necrosis/apoptosis with inflammatory cell infiltration, and typical areas of perivascular and interstitial replacement fibrosis are seen. Electrophysiological data further suggest that myocardial scar burden and distribution pattern differ significantly between ischemic and nonischemic chronic heart failure patients being significantly less in quantity and more diffuse in distribution in the latter group [6]. On subcellular level, abnormal shapes, sizes, and numbers of mitochondria have been reported in DCMP. The exact pathophysiological mechanisms that underlie these changes still remain poorly understood, and it is currently believed that the interplay between altered sarcomeric and cytoskeletal proteins, direct pathogen damage, postinfection immune and autoimmune mechanisms, and free oxygen radical species may represent the foundation of the development and progression of DCMP [5].

Additionally, defective vascularization with impaired vasculogenesis and angiogenesis has also been documented in patients with DCMP [7] suggesting that, in addition to myocardial inflammation, microvascular ischemia may represent one of the key mechanisms involved in the development of DCMP and its progression to end-stage heart failure. Currently, the mechanisms that lead to altered vasculogenesis and angiogenesis in DCMP remain largely unexplained. They appear to be related to impaired myocardial homing, mediated mainly through the SDF-1/CXCR4 axis, and survival of circulating CD34^+^ cells and endothelial progenitor cells (EPC) in the earlier phases (NYHA I and II) of the disease and to the exhaustion of the pools of these progenitors in the later phases (NYHA III and IV) of the disease [8–10].

## 3. Stem Cell Types

Generally, stem cells are defined as a population of self-renewing cells with the potential to generate daughter cells with the capability to differentiate along specific cell lineages [11]. In the last decade, several stem cell types have been studied for the treatment of HF, alone or in combination, and for the most part, the studies have focused on ischemic HF. Only a handful of clinical trials specifically addressed the stem cell therapy in the setting of DCMP (Table 1).

### 3.1. Bone Marrow-Derived Stem Cells (BMMC)

Bone marrow is the source of mixed population of hematopoietic and nonhematopoietic stem cells. Both have been shown to possess the ability to transdifferentiate into different cell lineages if transferred to a tissue-specific cytokine milieu. Due to the easy access and straightforward procurement, this stem cell type has understandably gained the widest attention in preclinical and early clinical trials. Mostly, BMMC were studied in the setting of ischemic HF. However, several studies did analyse the safety and efficiency of this stem cell population also in patients with DCMP.

In the TOPCARE-DCM study (a pilot trial assessing potential effects of selective intracoronary bone marrow-derived progenitor cell infusion on patients with nonischemic dilated cardiomyopathy; NCT00284713), intracoronary infusion of BMMC into the left anterior descending coronary artery was performed in 33 patients by an over-the-wire balloon catheter. This resulted in improved regional wall motion of the injected area and global left ventricular myocardial performance with an average increase in left ventricular ejection fraction (LVEF) by 3%. Furthermore at 12-month follow-up, NT-proBNP serum levels were persistently decreased suggesting a potential beneficial effect of BMMC infusion on LV remodeling process [12]. In the ABCD trial (percutaneous intracoronary cellular cardiomyoplasty for nonischemic cardiomyopathy; NCT unavailable), 44 patients with nonischemic HF received either intracoronary injection of BMMC (24 patients) or sham infusion without stem cells (20 patients). In the treatment arm, 2/3 of the cell suspension was infused in the left coronary system and 1/3 in the right coronary system [13]. During the 3-month follow-up, LVEF improved in the treatment arm by 5.4% but remained largely unchanged in the control arm, and similarly, improvement in NYHA functional class was observed in the treatment arm but not in controls [13]. Similarly, in a study of patients with refractory nonischemic HF, infusion of BMMC into the left main coronary artery was associated with improved myocardial performance, maximal oxygen consumption, and quality of life [14]. Recently, REGENERATE-DCM (randomized trial of combination cytokine and adult autologous bone marrow progenitor cell administration in patients with nonischemic dilated cardiomyopathy; NCT 01302171) corroborated these encouraging early results by showing a 5.4% increase in LVEF in 15 DCMP patients who received intracoronary infusion of BMMC suspension. This improvement in left ventricular function was additionally associated with a decrease in NYHA functional class, reduction in NT-proBNP serum levels, and increase in patient exercise capacity, all of which persisted over the 1-year follow-up period. Of note, this trial also explored possible effects of peripheral G-CSF stimulation on LVEF and found no correlation, discarding the argument that peripheral G-CSF stimulation itself is sufficient to promote homing and engraftment of circulating stem cells to the diseased myocardium [15].

Taken together, these data suggest that BMMC therapy may be of potential clinical benefit in patients with DCMP. However, the variations in BMMC populations, patient selection criteria, and delivery methods used in these studies make direct comparisons between them challenging and any extrapolation to a wider clinical utility very difficult.

### 3.2. Hematopoietic Stem Cells (HSC)

HSC represent a part of the hematopoietic cell compartment of the bone marrow that differentiates along lymphoid and myeloid lineages to form mature white blood cells. HSC are positive for the CD34^+^ surface marker and have been shown to have a potential to differentiate into endothelial cells and thus promote target tissue neovascularization [16] directly addressing one of the main pathophysiologic mechanisms involved in DCMP. Since in comparison to BMMC their procurement is more cumbersome and associated with more complex logistics and higher cost, it is not surprising that this stem cell population has not been extensively studied.

In the first clinical trial assessing the effects of stem cell therapy, 28 DCMP patients received intracoronary application of CD34^+^ stem cells obtained through G-CSF stimulation followed by peripheral blood apheresis and immunomagnetic selection. In comparison to the control group, a 5% increase in LVEF was observed in the stem cell group at 1-year follow-up. Additionally, improved functional capacity of the patients and reduced neurohumoral activation were observed [17]. Importantly, these positive effects persisted through the 5-year follow-up period and translated to improved survival of patients receiving CD34^+^ cell therapy [18]. Recently published data further suggest that transendocardial stem cell injections may be preferred to intracoronary stem cell infusion in this patient population. It was shown that transendocardial stem cell injections yielded 5 times higher retention rates (around 18%) than intracoronary stem cell infusion (around 4%) which in turn translated into better functional recovery of the left ventricle (LVEF improved by 8% in the transendocardial group versus 4% in the intracoronary group), better recovery of exercise capacity, and a more pronounced decrease in neurohumoral activation in this patient population [19]. Whether higher stem cell retention rates translate to additional survival benefit in patients with DCMP remains to be further explored. The currently ongoing REMEDIUM trial (repetitive intramyocardial CD34^+^ cell therapy in dilated cardiomyopathy; NCT 02248532) is evaluating the potential benefits of repetitive transendocardial CD34^+^ stem cell injections in DCMP patients, and the results are expected to be available by the end of 2017.

### 3.3. Mesenchymal Stem Cells (MSC)

MSC represent a part of the nonhematopoietic cell compartment of the bone marrow and have been reported to differentiate into cardiomyocytes [20] and endothelial cells [21] in vitro. The potential advantage of these cells over other stem cell types studied to date is that they are immunoprivileged and thus do not cause the activation of immune response and allosensitization, which enables them to be used in an allogeneic setting.

Two clinical trials explored MSC in DCMP. Butler et al. evaluated in a placebo-controlled crossover trial the safety and efficacy of intravenously applied allogeneic MSC (1.5 × 10^6^/kg) in 22 DCMP patients. Although at 90 days no improvement of LVEF was observed, the data suggested better exercise capacity and quality of life after stem cell treatment [22]. The larger POSEIDON-DCM trial (randomized comparison of allogeneic versus autologous mesenchymal stem cells for nonischemic dilated cardiomyopathy; NCT 01392625) compared the safety and efficiency of transendocardial injection of autologous and allogeneic MSC. The authors demonstrated significantly better response to allogeneic MSC compared to autologous MSC. The former was associated with better improvement in LVEF (8% versus 4%), decrease in myocardial inflammation, increase in patient exercise capacity, and quality of life [23]. Several factors might account for the greater efficacy of allogeneic MSC and include MSC donor age (the mean age in the allogeneic MSC group was about 50% of the autologous MSC group) and the possible adverse impact of the systemic proinflammatory milieu of heart failure on autologous MSC [23]. Despite the encouraging results of the study of Butler et al. and POSEIDON-DCM, larger trials are warranted to confirm these data. The currently ongoing phase III trial DREAM-HF (efficacy and safety of allogeneic mesenchymal precursor cells (Rexlemestrocel-L) for the treatment of heart failure; NCT 02032004) focusing on chronic heart failure of both ischemic and nonischemic etiologies will hopefully corroborate these data and firmly establish the allogeneic MSC therapy for the treatment of DCMP.

### 3.4. Cardiosphere-Derived Cells (CDC)

CDC represent a heterogeneous mix of cells, derived from myocardial biopsy tissue, and comprise of cells that express hematopoietic as well as mesenchymal antigens [24]. In vitro CDC were shown to differentiate into cardiomyocytes, and animal data suggested that intracoronary injections of CDCs may promote myocardial repair [25]. There are currently no complete clinical trials of CDC in DCMP. The ongoing DYNAMIC trial (the dilated cardiomyopathy intervention with allogeneic myocardially regenerative cells; NCT unavailable) is aiming to evaluate the safety of allogeneic CDC in DCMP patients, and the results of the trial are expected in 2020.

### 3.5. Autologous versus Allogeneic Stem Cells

In recent years, using allogeneic stem cell products became of significant interest in the field of heart failure management. Two obvious reasons for this shift in clinical practice are the standardization of stem cell products and the generation of “off-the-shelf” products where heart failure patients can be treated without prior bone marrow stimulation and/or aspiration which is invasive, costly, and logistically quite cumbersome.

The other, clinically more significant, reason is that sicker heart failure patients generate less stem cells that are also less potent [8–10]. Although underlying mechanisms remain incompletely explained, heart failure-associated systemic inflammatory response is currently believed to be the main culprit [9, 10]. Several studies, published in the last decade, also demonstrated that circulating EPC and CD34^+^ cells are reduced in the presence of cardiovascular risk factors (CVRF) such as arterial hypertension, diabetes, and hyperlipidemia [26]. Additionally nonmodifiable CVRF such as male gender and age were also associated with lower circulating EPC count [27]. It was further shown that clustering of these risk factors leads to progressive reduction in circulating stem cell count [28].

It was also established that in comparison to patients with ischemic heart disease, patients with DCMP express significantly less myocardial homing factors, further reducing the efficiency of endogenous repair mechanisms of the myocardium in this patient population [9].

To overcome these limitations of autologous stem cell therapy, allogeneic stem cells were recently explored [23, 29, 30]. Initial clinical experience is encouraging as allogeneic stem cells were demonstrated to be safe and feasible and may thus represent an important step towards standardization of stem cell therapy in patients with ischemic and nonischemic heart failure. Currently, larger trials are needed to firmly establish the efficiency of allogeneic stem cell therapy in patients with heart failure.

## 4. Delivery Methods

The ability of the damaged myocardium to attract circulating stem cells is fundamental to myocardial repair. It is currently suggested that acutely injured myocardium generates cytokine-mediated signals for the mobilization of stem cell pool from bone marrow to peripheral circulation. Afterwards, these circulating bone marrow-derived cells follow a SDF-1 cytokine gradient that enables them to home to the damaged regions of the myocardium [26]. However, in the setting of chronic heart failure, these stem cell recruitment and homing stimuli are significantly decreased, insufficient to significantly mitigate the myocardial injury, and actually favor the retention of stem cells in the bone marrow or in the peripheral circulation [9]. This limitation can be overcome by exogenous delivery of stem cells to the injured myocardium.

To date, no consensus has been reached with regard to the optimal mode of stem cell delivery to the failing myocardium. Intramyocardial (IM) approach and intracoronary (IC) approach have mainly been used in the clinical settings [27]. By far, the most widely clinically used approach is the IC stem cell delivery [28]. While being demonstrated to be simple, cheap, and safe, it has two major limitations: first, stem cells cannot reach the sites of the poorly perfused myocardium, thereby limiting the feasibility of this approach in patients with chronic ischemic HF who typically have diffused and advanced coronary artery disease. Secondly, using larger cell types and/or higher stem cell doses may cause microemboli and thus an obstruction of the target coronary artery causing additional ischemic damage to the already failing myocardium. IM route is the most aggressive among the approaches for stem cell therapy. However, especially when used in combination with electroanatomical mapping, it provides the most direct and precise stem cell delivery to the target myocardium. Although associated with higher costs and requiring additional training, it was demonstrated to be safe and efficient yielding significantly higher myocardial cell retention rates than IC stem cell delivery [19].

## 5. Efficiency of Stem Cell Therapy in Nonischemic Heart Failure

**Figure 1:**
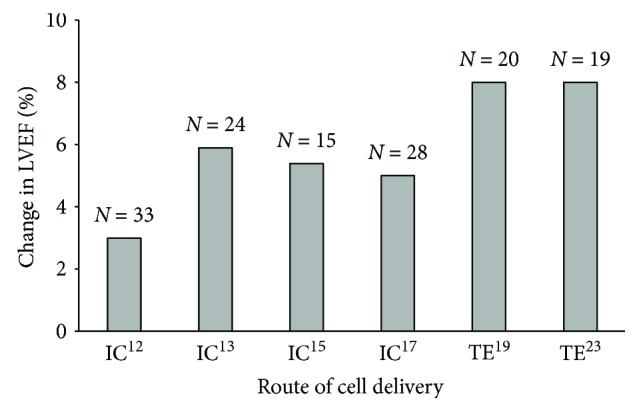
Clinical trials of stem cell therapy in patients with DCMP showed improvement in left ventricular ejection fraction with transendocardial delivery being seemingly more efficient. TE: transendocardial cell delivery; IC: intracoronary cell delivery.

Studies of stem cell therapy in patients with DCMP have demonstrated signs of improvement in LVEF (Figure 1) and exercise capacity [12, 13, 15, 17, 23]. Whether these improvements translate to improved outcomes (hospital readmissions, survival) of this patient cohort, however, remains largely unanswered.

Our unpublished RECORD registry (registry of cell therapy in nonischemic dilated cardiomyopathy; NCT02445534) data on 148 chronic HF patients suggest that stem cell therapy may lead to reduced hospital readmissions due to worsening heart failure. Within one year after stem cell therapy, we have observed a significant decrease in heart failure-related hospital readmissions when compared to the 1-year interval before cell therapy (0.8 ± 0.8 admission/year before cell therapy and 0.5 ± 0.9 admission/year after cell therapy; *P* = 0.007).

**Figure 2:**
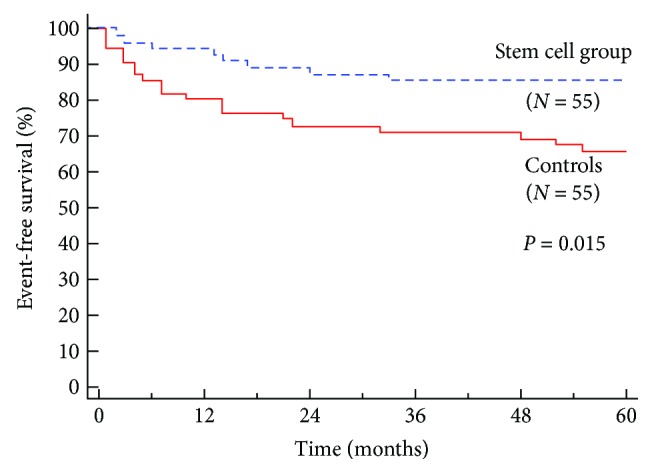
Kaplan-Meier survival plot of DCMP patients who received intracoronary CD34^+^ stem cell therapy (stem cell group) and patients treated with standard therapy (controls). Published with permission of Professor Bojan Vrtovec.

The longest follow-up of DCMP patients receiving stem cells published to date showed that after 5 years, patients who received CD34^+^ stem cells had significantly lower cardiovascular mortality being 14% in the stem cell group (55 patients) and 35% in controls (55 patients) (Figure 2). Additionally, pump failure, but not sudden cardiac death (9% versus 16%, *P* = 0.39) [18], was found to be significantly lower in the treatment arm (5% versus 18%, *P* = 0.03).

Although these results are based on small single-center data and need to be confirmed in larger trials, they still offer an encouraging signal that stem cell therapy on top of optimal medical management further improves long-term prognosis of DCMP patients.

## 6. Conclusion

In summary, the data of currently available clinical trials of stem cell therapy in DCMP have shown promising results regarding the improvement of LVEF, patients' functional capacity, and quality of life. This is in stark contrast to the clinical trials of stem cell therapy in ischemic heart disease that failed to consistently demonstrate the beneficial effect of this treatment modality, thus making DCMP an apparently more inviting target for stem cell therapy. Given the heterogeneity of clinical characteristics of patients with DCMP, it may be difficult to define a single “fit-all” stem cell therapeutic approach. Future stem cell strategies should aim for a more personalized therapeutic approach by establishing the optimal stem cell type or their combination, dose, and delivery method for an individual patient adjusted for underlying causes of heart failure and stage of the disease.

## Figures and Tables

**Table 1:** Clinical trials of stem cells in DCMP.

Study name	Study design	Study phase	Prespecified endpoint	Method of LVEF evaluation	Number of patients	Follow-up (months)	Cell type	Number of transplanted cells (million)	Cell delivery method
TOPCARE-DCM [12] (NCT00284713)	Prospective, open-label	II	(i) Absolute change in regional LV wall motion of the target area	LV angiography	33	12	BMMC	259 ± 135	IC

ABCD [13] (NCT unavailable)	Prospective, randomized, open-label	II	(i) Change in NYHA functional class (ii) Change in LVEF (iii) Mortality (iv) Histopathologic evaluation	Echo	44 (24 treated/20 controls)	6	BMMC	28 ± 16	IC

Bocchi et al. [14] (NCT unavailable)	Prospective, randomized, open-label	II	(i) Improvement in LVEF (ii) Exercise capacity (iii) QoL	Echo	40 (23 treated/17 controls)	1	BMMC	Not available	IC

REGENERATE-DCM [15] (NCT01302171)	Prospective, randomized, placebo-controlled, double-blind	II	(i) Change in global LVEF at 3 months (ii) Change in global LVEF at 12 months (iii) Exercise capacity (iv) QoL (v) Change in NT-proBNP	CMR/cCT	60 (15 peripheral G-CSF, 15 peripheral placebo, 15 IC stem cells, 15 IC serum)	12	BMMC	216 ± 221	IC

Vrtovec et al. [17] (NCT00629018)	Prospective, randomised, open-label	II	(i) Change in global LVEF at 12 months (ii) Exercise capacity (iii) Change in NT-proBNP	Echo	55 (28 treated/27 controls)	12	HSC (CD34^+^)	123 ± 23	IC

Vrtovec et al. [18] (NCT01350310)	Prospective, randomised, open-label	II	(i) Change in global LVEF at 12 months (ii) Exercise capacity (iii) Change in NT-proBNP	Echo	110 (55 treated/55 controls)	60	HSC (CD34^+^)	113 ± 26	IC

Vrtovec et al. [19] (no NCT number)	Prospective, randomised, open-label	II	(i) Change in global LVEF at 12 months (ii) Exercise capacity (iii) Change in NT-proBNP	Echo	40 (20 IC injections/20 TE injections)	6	HSC (CD34^+^)	IC: 103 ± 27 TE: 105 ± 31	IC/TE

Butler et al. [22] (NCT02467387)	Prospective, randomized, single-blind, placebo-controlled	II	(i) All-cause mortality (ii) All-cause hospitalizations (iii) Adverse events (iv) LVEF (v) Exercise capacity (vi) QoL (vii) Change in NT-proBNP	CMR	22 (10 treated/12 placebo)	6	alloMSC	1.5 million cells/kg	IV

POSEIDON-DCM [23] (NCT01392625)	Prospective, randomised, open-label	I/II	(i) Adverse events (ii) LVEF (iii) Exercise capacity (iv) QoL	CMR/cCT/echo	37 (19 alloMSC/18 autoMSC)	12	AlloMSC versus autoMSC	100	TE

BMMC: bone marrow mononuclear cells; HSC: hematopoietic stem cells; alloMSC: allogeneic mesenchymal stem cells; autoMSC: autologous mesenchymal stem cells, IC: intracoronary route; TE: transendocardial route; IV: intravenous route; QoL: quality of life.
